# Student's Knowledge, Attitudes, and Practices Related to Cardiopulmonary Resuscitation at Qassim University, Saudi Arabia

**DOI:** 10.7759/cureus.6169

**Published:** 2019-11-16

**Authors:** Ali Mansour, Ahmad H Alsager, Abdulrahman Alasqah, Abdullah S Altamimi, Abdulaziz Alsuhaibani, Ahmed A Aljabr, Abdullah I AlDughaim

**Affiliations:** 1 Family & Community Medicine, Unaizah College of Medicine, Qassim University, Unaizah, SAU; 2 Medicine, Unaizah College of Medicine, Qassim University, Unaizah, SAU

**Keywords:** cpr, knowledge, attitudes, practices, students, qassim

## Abstract

Background

Cardiopulmonary resuscitation (CPR) is a lifesaving technique that is vital to deal with in many emergencies. Early interference with the cardiopulmonary resuscitation technique is really important for the survival of the patient. This study assesses the level of knowledge, attitudes, and practices regarding CPR among Qassim University students.

Materials and methods

A cross-sectional study was conducted at Qassim University. The sample was university students. The colleges were chosen by a simple random technique. Data were collected using a pretested, semi-structured questionnaire for knowledge, attitudes, and practices related to CPR. The data were analyzed using Statistical Package for the Social Sciences (SPSS; IBM Corporation, Armonk, NY).

Results

The total sample size of the participating students was 1148, of which 589 (51.3%) were female and the remaining were male (559, 48.7%). The common age was between 20 and 23 years old and most were from non-medical colleges (54.1%); the remaining (45.9%) were from medical colleges. The majority of female students (45.7%) in medical colleges knew the right location for chest compression better than the males, with a significant difference (p<0.05). No significant difference existed between males and females (p>0.05) regarding most CPR practices. On the other hand, there was a significant increase among medical students as compared to non-medical students (p<0.05) regarding most knowledge, attitudes, and practice items of CPR.

Conclusion

Based on our study, the knowledge, attitudes, and practices of Qassim University students toward CPR are insufficient and need to be improved. Also, medical students are better than non-medical students at CPR, so we recommend that the incorporation of a Basic Life Support (BLS) course, including CPR learning procedures in the university curriculum, with regular reassessments, would increase the knowledge and application of CPR skills among students for saving people's life.

## Introduction

Cardiopulmonary resuscitation (CPR) is the first prompt and initial action provided to a cardiac arrest patient. In the best possible condition, this efficient action is taken to keep the patient alive until emergency medical services and treatment can be obtained. Early intervention with the CPR technique is crucially important for the survival of the patient. The more effective and efficient the caregiver or ‘bystander’ is, the better the results will be on the ground [1].

Cardiac arrest is the sudden stopping of the pumping function of the heart and, therefore, the need for CPR for people whose hearts or breathing has stopped. The mechanism of CPR is performed by doing rescue breathing and chest compressions. It is known that about 75% of cardiac arrests happen at home. Therefore, the need for CPR by bystanders emerges to raise the chances of survival of the patient. Studies have shown that immediate and successful CPR may raise the chance of survival from double to triple [2-3].

Sudden cardiac death (SCD) is a common and devastating event, often occurring in the early age of one's life. SCD is considered to be the most common and leading cause of death outside the hospital worldwide. In the USA, according to one study, the incidence of SCD was found to be 300,000-450,000 per year. Researchers also found that the peaks of SCD are bimodal, occurring at the age of infancy and after the age of 45. In the USA, almost 50% of adults who suffer from SCD were found to have coronary heart disease (CHD). One study was done on New Zealand's high-school students to assess the level of the knowledge of the students towards CPR. The students showed a poor understanding of resuscitation; only 22% of them knew what CPR means [4-5].

According to one study on King Saud University students in Riyadh, Saudi Arabia, it found that 31% of the students had no idea about CPR, 85% felt that their knowledge was weak, and 10% believed that their knowledge was good [6].

According to our search, no study was done regarding the knowledge and attitudes towards CPR that has included all Qassim University students in the Qassim region of Saudi Arabia. So, our study was conducted to assess the level of knowledge, attitudes, and practices regarding cardiopulmonary resuscitation among all Qassim University students.

## Materials and methods

Study design

A cross-sectional study was conducted during the academic year 2018-2019.

Study setting

Students of Qassim University, Qassim region, Saudi Arabia

Sample size

The study participants are samples from the students of Qassim University. The colleges were chosen by the simple random technique. The sample size was calculated by using a specific sample size equation for a cross-sectional study: n = Z² (1-α) p (1-p) /d² The total sample size was 1148 students from seven colleges.

Sampling technique

The simple random technique

Data collection methods

Data were collected using a pretested, semi-structured questionnaire. The questionnaire was devised from the literature and formal discussions with experts. It is in the Arabic language, and it consisted of 37 items pertaining to the demographic profile, knowledge, attitudes, and practices towards CPR among university students at Qassim University, Saudi Arabia.

Pilot study

The pilot study was done on 20 students (10 male and 10 female) before starting our study, to test the validity of our questionnaire and to estimate the timing needed for each participant to complete the questionnaire.

Data analysis plan

The data were entered, organized, tabulated, and analyzed by using Statistical Package for the Social Sciences (SPSS) version 24 (IBM Corporation, Armonk, NY) and for the purpose of data analysis and presentation. Qualitative data were expressed as frequency and percent. Chi-square (x2) was used to assess the relationship between the qualitative variables and the Students t-test was used for comparing the quantitative variables with the significance level set at a p-value of <0.05.

## Results

Characteristics of participant students

The total sample size of the participating students was 1148, of which 589 (51.3%) were female and the remaining were male (559; 48.7%). The common age for them was between 20 and 23 years old. Most of them were from non-medical colleges (54.1%) and the remaining (45.9%) from medical colleges. Also, the majority of students were in the third and fourth academic years (28.7% and 22.4%, respectively). The family income of most students was more than 10000 SR (≈ 2666 USD) per month (Table 1).

**Table 1:** Demographic characteristics of participating students

Demographic characteristics		Frequency N=1148	Percent
Gender	Male	559	48.7
	Female	589	51.3
Age	<20 years	105	9.1
	20-23 years	898	78.2
	>23 years	145	12.6
Colleges	Unaizah College of Medicine	294	25.6
	Unaizah College of Pharmacy	156	13.6
	Unaizah College of Sciences and Arts	229	19.9
	College of Sharia	105	9.1
	College of Engineering	78	6.8
	College of Applied Health Sciences in Al Rass	79	6.9
	College of Sciences and Arts in Abadyaa	207	18.0
Academic Years	1^st^ year	126	11
	2^nd^ year	212	18.5
	3^rd^ year	329	28.7
	4^th^ year	257	22.4
	5^th^ year	224	19.5
Family Income	≤ 10000 SR	403	35.1
	> 10000 SR	745	64.9
Medical vs. Non- Medical colleges	Medical college students	527	45.9
	Non-medical college students	621	54.1
Total		1148	100

Students' knowledge of CPR

The majority of students knew the emergency telephone number of Saudi Arabia Red Crescent (55.1%) and only (35.6%) of them knew the abbreviation of BLS. In addition, 43.0% of students mentioned that the survival rate in out-of-hospital cardiac arrest if CPR is performed correctly is 70%. Most of them (44.1%) revealed that the mid-chest is the location of chest compression application and 40.8% of them stated that 2½ inches to 3 inches is the proper chest compression depth in CPR for adult persons. Only 24.2% of students mentioned that the chest compression rate for adults and children is 100/min. Also, 36.3% of them revealed that the chest compression rate in CPR if you are the only one doing it is 5:1 (Table 2).

**Table 2:** Knowledge of Qassim University students regarding cardiopulmonary resuscitation (CPR)

Knowledge's items		Frequency N=1148	Percent
Do you know the emergency telephone number in Saudi Arabia “Red Crescent"?	Yes	632	55.1
	No	516	44.9
The abbreviation of BLS means?	Best Life Support	247	21.5
	Basic Life Support	409	35.6
	Basic Lung Support	277	24.1
	Basic Life Services	215	18.7
What is the survival rate in out-of-hospital cardiac arrest if CPR is performed correctly?	1%	30	2.6
	25%	179	15.6
	70%	494	43.0
	95%	445	38.8
Where is the location of chest compression application?	Left side of the chest	210	18.3
	Right side of the chest	71	6.2
	Mid chest	506	44.1
	Xiphisternum	361	31.4
What is the proper chest compression depth in CPR for an adult person?	2½ – 3 inches	468	40.8
	1½ – 2 inches	417	36.3
	1– 1½inches	170	14.8
	½ – 1 inch	93	8.1
What is the chest compression rate in CPR? For adult and child persons?	120/mint.	251	21.9
	100/mint.	278	24.2
	80/mint.	254	22.1
	70/mint.	365	31.8
What is the chest compression rate in CPR? If you are the only one to do that?	30:2	266	23.2
	15:2	320	27.9
	15:1	145	12.6
	5:1	417	36.3
Total		1148	100.0

Attitudes of students towards CPR

About 55.5% of students think that a CPR training course is mandatory for all students as a graduation requirement, and about 40.1% of them stated that the best method to increase public awareness of the importance of CPR is to inform people about the currently available training courses. The majority of students strongly agree and also agree about the benefits of CPR training courses to public life saving (66.9% and 24.9%, respectively). Also, most students (77.5%) did not have a CPR training course, and the majority of them (82.5%) wanted to learn CPR (Table 3).

**Table 3:** Attitudes of Qassim University students regarding cardiopulmonary resuscitation (CPR)

Attitudes items		Frequency N=1148	Percent
Do you think CPR training courses should be:	Mandatory for all students (graduation requirement)	635	55.3
	Mandatory for some majors	205	17.9
	Optional	269	23.4
	Don’t support the implementation of training courses	39	3.4
What is the BEST method-in your opinion- to increase public awareness of the importance of CPR?	Increased publicity	415	36.1
	Inform people of the training courses currently available	466	40.6
	Free training courses	176	15.3
	Increase the number of courses	80	7.0
	Others	11	1.0
Do you think CPR training is a benefit for students to save the lives of the public?	Strongly agree	768	66.9
	Agree	286	24.9
	Neutral	79	6.9
	Disagree	8	0.7
	Strongly disagree	7	0.6
Have you ever taken a CPR training course?	Yes	258	22.5
	No	890	77.5
What encouraged you to take the course?	Work or graduation requirement (Mandatory)	57	5.0
	Personal benefit (Optional(	179	15.6
	Previous experience proved the importance of CPR	16	1.4
Do you want to learn CPR?	Yes	947	82.5
	No	201	17.5

Practices of students towards CPR

With regards to the first response of students when they saw a comatose person, most of them stated that they looked for safety and open airways (46.4% and 35.5%, respectively). The majority of students would activate emergency medical services (EMS) and start CPR if someone did not respond by moving or talking (47.9% and 35.8%, respectively). Of the students, 34.4% would do mouth-mask ventilation and chest compression during CPR if they did not want to give artificial respiration. Of the participants, 37.9% will give abdominal thrusts to their friends if they were exposed to choking during eating. About 50% of students would do back blows and chest compression for five cycles and then open the mouth and remove the foreign body if they watched a child who suddenly started to choke. Of the participants, 42.9% would compress the abdomen to remove water for a drowned person. Of the students, 43.5% would take their colleagues to the nearest clinic if they were not able to speak and exposed to weakness in the right upper limb (Table 4).

**Table 4:** Practices of Qassim University students regarding cardiopulmonary resuscitation (CPR) EMS: emergency medical services

Practices items		Frequency N=1148	Percent
If you see someone comatose, what is your first response?	Open airway	409	35.6
	Start chest compression	163	14.2
	Look for safety	533	46.4
	Give two breathings	43	3.7
After making sure that someone does not respond to you even after you try to move him and talk to him what to do	Start CPR	411	35.8
	Activate EMS	550	47.9
	Put him in the recovery position	148	12.9
	Observe	39	3.4
If you do not want to give artificial respiration by direct contact with the mouth during CPR work, you can do the following:	Mouth-mask ventilation and chest compression	399	34.4
	Chest compression only	264	23.0
	Mask ventilation with chest compression	194	16.9
	No CPR	291	25.3
If you and your friend are eating at the canteen and suddenly your friend is exposed to the symptoms of choking what to do	Give abdominal thrusts	435	37.9
	Give chest compression	243	21.2
	Confirm foreign body aspiration by talking to him	154	13.4
	Give back blows	316	27.5
You are watching a child who suddenly started to choke while playing with him. I have made sure he is unable to cry or cough. What is your first response?	Start CPR immediately	122	10.6
	Try to remove the suspected foreign body by blind finger sweeping technique	395	34.4
	Back blows and chest compression of five cycles each then open the mouth and remove foreign body only when it is seen	569	49.6
	Give water to the infant	62	5.4
You see a person who does not respond after drowning in water. After taking him out, he has breathing but does not respond. What is the first step	CPR for two minutes and inform EMS	297	25.9
	CPR for one minute and inform EMS	239	20.8
	Compress the abdomen to remove the water	492	42.9
	Keep him in the recovery position	120	10.5
You noticed that your colleague was not able to speak and he exposed to weakness in the right upper limb which one of the following could be done	Offer him some drinks, probably hypoglycemia	307	26.7
	Possibly stroke, get him to the nearest clinic	499	43.5
	Possibly stroke, he may require thrombolysis and hence activate emergency medical services	244	21.3
	May be due to sleep deprivation, make him sleep.	98	8.5

Gender comparison for knowledge and attitudes

Male students (58.3%) knew the emergency telephone for Saudi Red Crescent more than females did, but the majority of female students (45.7%) knew the right location for the application of chest compression more than males did, with a significant difference between them (p<0.05) and 43.6% of female participants knew the proper depth of chest compression and the rate of CPR (23.9%) more than males did, with no significant difference between them (p>0.05). The majority of female students thought that the CPR training course is mandatory for all students as a graduation requirement (62.1%), and they mentioned that the best method, in their opinion, to increase the awareness of the public regarding the CPR training course is to inform people of the training courses currently available (44.8%). They also strongly agreed that the CPR training course is beneficial for students to save the lives of the public (73.7%). And most of them (86.6%) wanted to learn CPR more than males did, with a significant difference between them (p<0.05) (Table 5).

**Table 5:** Comparison between genders regarding the knowledge and attitudes of cardiopulmonary resuscitation (CPR) *Significant

Variables		Male (n= 559) No %	Female (n=589) No %	P-value
Do you know the emergency telephone number in Saudi Arabia “Red Crescent"?	Yes	326(58.3%)	306(52.0%)	0.06
	No	233(41.7%)	283(48.0%)	
The abbreviation of BLS. Means?	Best Life Support	111(19.9%)	136(23.1%)	<0.001 S*
	Basic Life Support	177(31.7%)	232(39.4%)	
	Basic Lung Support	136(24.3%)	141(23.9%)	
	Basic Life Services	135(24.2%)	80(13.6%)	
What is the survival rate in out-of-hospital cardiac arrest if CPR is performed correctly?	1%	19(3.4%)	11(1.9%)	<0.001 S*
	25%	111(199%)	68(11.5%)	
	70%	251(44.9%)	243(41.3%)	
	95%	178(31.8%)	267(45.3%)	
Where is the location of the chest compression application?	Left side of the chest	96(17.2%)	114(19.4%)	0.016 S*
	Right side of the chest	47(8.4%)	24(4.1%)	
	Mid chest	237(42.4%)	269(45.7%)	
	Xiphisternum	179(32.0%)	182(30.9%)	
What is the proper chest compression depth in CPR For adult person?	2½ – 3 inches	211(37.7%)	257(43.6%)	0.236
	1½ – 2 inches	215(38.5%)	202(34.3%)	
	1– 1½inches	87(15.6%)	83(14.1%)	
	½ – 1 inch	46(8.2%)	47(8.0%)	
What is the chest compression rate in CPR for adults and children?	120/mint.	114(20.4%)	137(23.3%)	0.096
	100/mint.	137(24.5%)	141(23.9%)	
	80/mint.	140(25.0%)	114(19.4%)	
	70/mint.	168(30.1%)	197(33.4%)	
What is the chest compression rate in CPR., If you are the only one to do that?	30:2	134(24.0%)	132(22.4%)	0.564
	15:2	146(26.1%)	174(29.5%)	
	15:1	69(12.3%)	76(12.9%)	
	5:1	210(37.6%)	207(35.1%)	
Do you think CPR training courses should be:	Mandatory for all students (graduation requirement)	269(48.1%)	366(62.1%)	<0.001 S*
	Mandatory for some majors	121(21.6%)	84(14.3%)	
	Optional	145(25.9%)	124(21.1%)	
	Don’t support the implementation of training courses	24(4.3%)	15(2.5%)	
What is the BEST method-in your opinion- to increase public awareness of the importance of CPR?	Increased publicity	229(41.0%)	186(31.6%)	<0.001 S*
	Inform people of the training courses currently available	202(36.1%)	264(44.8%)	
	Free training courses	76(13.6%)	100(17.0%)	
	Increase the number of courses	52(9.3%)	28(4.8%)	
	Others	0(0%)	11(1.9%)	
Do you think CPR training is beneficial for students to life-saving the public?	Strongly agree	334(59.7%)	434(73.7%)	<0.001 S*
	Agree	159(24.4%)	127(21.6%)	
	Neutral	55(9.8%)	24(4.1%)	
	Disagree	6(1.1%)	2(0.3%)	
	Strongly disagree	5(0.9%)	2(0.3%)	
Have you ever taken a CPR training course?	Yes	110(19.7%)	148(25.1%)	0.027 S*
	No	449(80.3%)	441(74.9%)	
What encouraged you to take the course?	Work or graduation requirement (Mandatory)	30(28.3%)	27(18.5%)	0.064
	Personal benefit (Optional(	67(63.2%)	112(76.7%)	
	Previous experience proved the importance of CPR	9(8.5%)	7(4.8%)	
Do you want to learn CPR?	Yes	437(78.1%)	510(86.6%)	<0.001 S*
	No	122(21.8%)	79(13.4%)	

Gender comparison for practices

In regards the practice of CPR, there is no significant difference between males and females (p>0.05) in their first response if they saw a comatose person, giving abdominal thrusts if their friend was exposed to choking during eating, and compressing the abdomen to remove water if they saw a person drowning and not responding. But there is a significant increase among females rather than males (p<0.05) regarding the activation of EMS, starting CPR for someone not responding to moving and talking (48.0%), implementing back blows and chest compression of five cycles followed by opening the mouth and removing the foreign body on watching a child who suddenly started to choke (50.3%), and getting their colleagues to the nearest clinic if they were not able to speak and were exposed to weakness in the right upper limb (46.2%) (Table 6).

**Table 6:** Comparison between gender regarding practices for cardiopulmonary resuscitation (CPR) *Significant EMS: emergency medical services

Variables		Male (n=559) No %	Female (n=589) No %	P value
If you see someone comatose, what is your first response?	Open airway	214(38.3%)	195(33.1%)	0.158
	Start chest compression	81(14.5%)	82(13.9%)	
	Look for safety	241(43.1%)	292(49.6%)	
	Give two breathings	23(4.1%)	20(3.4%)	
After making sure that someone does not respond to you even after you try to move him and talk to him, what to do?	Start CPR	201(36.0%)	210(35.7%)	0.039 S*
	Activate EMS	267(47.8%)	283(48.0%)	
	Put him in recovery position	64(11.4%)	84(14.3%)	
	Observe	27(4.8%)	12(2.0%)	
If you do not want to give artificial respiration by direct contact with the mouth during CPR work, you can do the following:	Mouth-mask ventilation and chest compression	190(34.0%)	209(35.5%)	0.100
	Chest compression only	115(20.6%)	149(25.3%)	
	mask ventilation with chest compression	106(19.0%)	88(14.9%)	
	No CPR	148(26.5%)	143(24.3%)	
If you and your friend are eating at the canteen and suddenly your friend is exposed to the symptoms of choking, what to do?	Give abdominal thrusts	203(36.3%)	232(39.4%)	0.153
	Give chest compression	118(21.1%)	125(21.2%)	
	Confirm foreign body aspiration by talking to him	68(12.2%)	86(14.6%)	
	Give back blows	170(30.4%)	146(24.8%)	
You are watching a child who suddenly started to choke while playing with him. I have made sure he is unable to cry or cough. What is your first response?	Start CPR immediately	71(12.7%)	51(8.7%)	0.003 S*
	Try to remove the suspected foreign body by blind finger sweeping technique	175(31.3%)	220(37.4%)	
	Back blows and chest compression of five cycles each then open the mouth and remove foreign body only when it is seen	273(48.8%)	296(50.3%)	
	Give water to the infant	40(7.2%)	22(3.7%)	
You see a person who does not respond drowned in the water. After getting out, he is breathing but does not respond. What is the first step?	CPR for two minutes and inform EMS	147(26.3%)	150(25.5%)	0.632
	CPR for one minute and inform EMS	108(19.3%)	131(22.2%)	
	Compress the abdomen to remove the water	242(43.3%)	250(42.4%)	
	Keep him in the recovery position	62(11.1%)	58(9.8%)	
You noticed that your colleague was not able to speak and he was exposed to weakness in the right upper limb. Which one of the following could be done?	Offer him some drinks, probably hypoglycemia	141(25.2%)	166(28.2%)	0.003 S*
	Possibly stroke, get him to the nearest clinic	227(40.6%)	272(46.2%)	
	Possibly stroke, he may require thrombolysis and hence activate emergency medical services	128(22.9%)	116(19.7%)	
	May be due to sleep deprivation, make him sleep.	63(11.3%)	35(5.9%)	

Medical and non-medical comparison of knowledge and attitudes

There was a significant increase among medical students rather than non-medical students (p<0.05) regarding knowledge and attitude for knowing the emergency telephone of Saudi Red Crescent (61.1%), proper chest compression depth (44.2%), and their thinking that a CPR training course is mandatory for all students as a graduation requirement (66.4%). Their opinion regarding the best methods for increasing the awareness of the public about CPR was to inform people of the training courses currently available (42.9%). They strongly agreed that a CPR training course is beneficial for students to life-save the public (78.6%); 87.9% of them wanted to learn CPR. But there is no significant difference between medical and non-medical students (p>0.05) regarding the right location for the application of chest compression and the chest compression rate (Table 7).

**Table 7:** Comparison between medical and non-medical colleges regarding the knowledge and attitudes of cardiopulmonary resuscitation (CPR) *Significant

Variables		Medical n=527 No %	Non-medical n= 620 No %	P-value
Do you know the emergency telephone	Yes	322(61.1%)	310(49.9%)	0.001 S*
number in Saudi Arabia “Red Crescent"?	No	205(38.9%)	310(49.9%)	
The abbreviation of BLS means?	Best Life Support	87(16.5%)	160(25.8%)	0.000 S*
	Basic Life Support	249(47.2%)	160(25.8%)	
	Basic Lung Support	105(19.9%)	172(27.7%)	
	Basic Life Services	86(16.3%)	129(20.8%)	
What is the survival rate in out-of-hospital cardiac arrest if CPR is performed correctly?	1%	5(0.9%)	25(4.0%)	0.000 S*
	25%	70(13.3%)	109(17.6%)	
	70%	225(42.7%)	269(43.3%)	
	95%	227(43.1%)	218(35.1%)	
Where is the location of chest compression application?	Left side of the chest	92(17.5%)	118(19.0%)	0.147
	Right side of the chest	24(4.6%)	47(7.6%)	
	Mid chest	239(45.4%)	267(43.0%)	
	Xiphisternum	172(32.6%)	189(30.4%)	
What is the proper chest compression depth in CPR for adult persons?	2½ – 3 inches	233(44.2%)	235(37.8%)	0.000 S*
	1½ – 2 inches	200(38.0%)	217(34.9%)	
	1– 1½inches	69(13.1%)	101(16.3%)	
	½ – 1 inch	25(4.7%)	68(11.0%)	
What is the chest compression rate in CPR? For adult and child persons?	120/mint.	110(20.9%)	141(22.7%)	0.393
	100/mint.	138(26.2%)	140(22.5%)	
	80/mint.	120(22.8%)	134(21.6%)	
	70/mint.	159(30.2%)	206(33.2%)	
What is the chest compression rate in CPR? If you are the only one to do that?	30:2	161(30.6%)	105(16.9%)	0.000 S*
	15:2	127(24.1%)	193(31.1%)	
	15:1	69(13.1%)	76(12.2%)	
	5:1	170(32.3%)	247(39.8%)	
Do you think CPR training courses should be:	Mandatory for all students (graduation requirement)	350(66.4%)	285(45.9%)	0.000 S*
	Mandatory for some majors	96(18.2%)	109(17.6%)	
	Optional	67(12.7%)	202(32.5%)	
	Don’t support the implementation of training courses	14(2.7%)	25(4.0%)	
What is the BEST method, in your opinion, to increase public awareness of the importance of CPR?	Increased publicity	222(42.1%)	193(31.1%)	0.000 S*
	Inform people of the training courses currently available	226(42.9%)	240(38.6%)	
	Free training courses	52(9.9%)	124(20.0%)	
	Increase the number of courses	27(5.1%)	53(8.5%)	
	Others	0(0.0%)	11(1.8%)	
Do you think CPR training is beneficial for students for life-saving the public?	Strongly agree	414(78.6%)	354(57.0%)	0.000 S*
	Agree	84(15.9%)	202(32.5%)	
	Neutral	25(4.7%)	54(8.7%)	
	Disagree	2(0.4%)	6(1.0%)	
	Strongly disagree	2(0.4%)	5(0.8%)	
Have you ever taken a CPR training course?	Yes	125(23.7%)	133(21.4%)	0.352
	No	402(76.3%)	488(78.6%)	
What encouraged you to take the course?	Work or graduation requirement (Mandatory)	28(23.1%)	29(22.1%)	0.683
	Personal benefit (Optional(	87(71.9%)	92(70.2%)	
	Previous experience proved the importance of CPR	6(5.0%)	10(7.6%)	
Do you want to learn CPR?	Yes	463(87.9%)	484(77.9%)	0.000 S*
	No	64(12.1%)	137(22.1%)	

Medical and non-medical comparison of practices

As regards the practice of CPR among medical and non-medical students. there is a significant increase among medical rather than than non-medical students (p<0.05) regarding their first response if they saw a comatose person (36.8%) and for giving abdominal thrusts if their friend was exposed to choking during eating (45.2%), the activation of EMS and starting CPR for someone not responding to moving and talking, implementing back blows and chest compression of five cycles, opening the mouth and removing the foreign body on seeing a child who suddenly started to choke (58.1%), and getting their colleagues to the nearest clinic if they not able to speak and were exposed to weakness in the right upper limb (51.8%). But there is an increased significance of non-medical rather than medical students for compressing the abdomen to remove water if they saw any person drowning and not responding (46.2%) (Table 8).

**Table 8:** Comparison between medical and non-medical colleges regarding practices for cardiopulmonary resuscitation (CPR) *Significant EMS: emergency medical services

Variables		Medical n=527 No %	Non-medical n= 620 No %	P-value
If you see someone comatose, what is your first response?	Open airway	194(36.8%)	215(34.6%)	0.001 S*
	Start chest compression	53(10.1%)	110(17.7%)	
	Look for safety	264(50.1%)	269(43.3%)	
	Give two breathings	16(3.0%)	27(4.3%)	
After making sure that someone does not respond to you even after you try to move him and talk to him, what to do?	Start CPR	211(40.0%)	200(32.2%)	0.000 S*
	Activate EMS	248(47.1%)	302(48.6%)	
	Put him in the recovery position	61(11.6%)	87(14.0%)	
	Observe	7(1.3%)	32(5.2%)	
If you do not want to give artificial respiration by direct contact with the mouth during CPR, you can do the following:	Mouth-mask ventilation and chest compression	169(32.1%)	230(37.0%)	0.000 S*
	Chest compression only	83(15.7%)	181(29.1%)	
	mask ventilation with chest compression	89(16.9%)	105(16.9%)	
	No CPR	186(35.3%)	105(16.9%)	
If you and your friend are eating at the canteen and suddenly your friend is exposed to the symptoms of choking what to do	Give abdominal thrusts	238(45.2%)	197(31.7%)	0.000 S*
	Give chest compression	104(19.7%)	139(22.4%)	
	Confirm foreign body aspiration by talking to him	83(15.7%)	71(11.4%)	
	Give back blows	102(19.4%)	214(34.5%)	
You are watching a child who suddenly started to choke while playing with him. I have made sure he is unable to cry or cough. What is your first response?	Start CPR immediately	43(8.2%)	79(12.7%)	0.000 S*
	Try to remove the suspected foreign body by blind finger sweeping technique	159(30.2%)	236(38.0%)	
	Back blows and chest compression of five cycles each then open the mouth and remove foreign body only when it is seen	306(58.1%)	263(42.4%)	
	Give water to the infant	19(3.6%)	43(6.9%)	
You see a person who does not respond after drowning in water. After taking him out, he is breathing but does not respond. What is the first step?	CPR for two minutes and inform EMS	167(31.7%)	130(20.9%)	0.001 S*
	CPR for one minute and inform EMS	105(19.9%)	134(21.6%)	
	Compress the abdomen to remove the water	205(38.9%)	287(46.2%)	
	Keep him in the recovery position	50(9.5%)	70(11.3%)	
You noticed that your colleague was not able to speak and he exposed to weakness in the right upper limb which one of the following could be done	Offer him some drinks, probably hypoglycemia	103(19.5%)	204(32.9%)	0.000 S*
	Possibly stroke, get him to the nearest clinic	273(51.8%)	226(36.4%)	
	Possibly stroke, he may require thrombolysis and hence activate emergency medical services	119(22.6%)	125(20.1%)	
	May be due to sleep deprivation, make him sleep.	32(6.1%)	66(10.6%)	

## Discussion

Cardiopulmonary resuscitation (CPR) is a lifesaving technique useful in many emergencies, including a heart attack or near drowning, in which someone's breathing or heartbeat has stopped. The American Heart Association recommends that everyone - untrained bystanders and medical personnel alike - begin CPR with chest compressions. It's far better to do something than to do nothing at all if you're fearful that your knowledge or abilities aren't 100% complete. Remember, the difference between your doing something and doing nothing could be someone's life [6].

The current study aimed to assess the level of knowledge, attitudes, and practices regarding cardiopulmonary resuscitation among Qassim University students.

The present study finds that around half of students don't know the emergency telephone of Saudi Arabia Red Crescent (45%). This is agreeing with another study conducted at Jouf University, which stated the same results. But it is less than another study conducted at King Saud University, which revealed that 70% of their students knew the Red Crescent telephone number [5,7].

On the other hand, this study showed that only 44% of students knew the right location of chest compression in CPR and this is in accordance with another study conducted at Qassim University, which revealed nearly the same results (40.0%) [8].

Furthermore, this study showed that only (40.8% and 24.2%, respectively) of students have insufficient knowledge regarding the proper chest compression depth in CPR and the chest compression rate for adults and children and this is due to the small number of training programs for CPR skills conducted at Qassim University. Also, there is no inclusion of a BLS course in the university curriculum for comparing with other students at King Saud University, which revealed sufficient knowledge 69% and 55%, respectively. However, this result is nearly the same as another study conducted among secondary school students in Riyadh and has better knowledge than another study conducted at Qassim University in 2014 [8-10].

As regards the attitudes of students towards CPR, the majority of students in this study (55.5%) think that the training program for CPR is mandatory for graduation, and it benefits them in saving the lives of people (66.9%). These results are better than another study conducted on university students in Riyadh, 2008, which found that only 45% of their students believe that it is mandatory for graduation [5].

On the other hand, the majority of students in this study wanted to learn CPR (82.5%), and this is a good, positive attitude among them. Also, this result is in accordance with another study conducted in New Zealand, which found nearly the same results (73.5%) and with a study that was conducted in Riyadh, 2008, which showed better attitudes (90%). These results reflect the importance of a CPR training program for university students and their benefit to them. The majority of students in our study think that the best way to increase the awareness of the public towards CPR is to inform them about the availability of training programs (40.1%). These results disagree with another study conducted in Riyadh, which shows that about half of the students believe that the media such as television and social media is the best way to increase the awareness of people [5,11].

Regarding the practice of students towards CPR, only 46.4% and 35.5% respectively, would look for safety and open airways in comatose persons. And this is insufficient practice for saving lives; this result is supported by another study conducted in 2014 at Qassim University, which revealed that the first practice for a comatose person is to look for safety and then an open airway. Furthermore, our study stated that the majority of students (47.9%) would activate EMS if they saw someone does not respond to moving or talking, and these results are in accordance with the same study conducted in 2014 [8].

On the other hand, this study stated that only 37.9% of participants would give abdominal thrusts to their friends if they are exposed to choking during eating. This is less than in another study conducted in 2010, which shows a higher level of practice (54.1%). Also, the present study finds that 50% of students will do back blows and chest compressions of five cycles, open the mouth, and remove the foreign body if they are watching a child who suddenly started to choke, and about 42.9% of the participants will compress the abdomen to remove water in a drowned person. This is in accordance with another study done in 2015 at an Ethiopian university. Furthermore, the current study shows that about 43.5% of students will take their colleagues to the nearest clinic if they were not able to speak and exposed to weakness in the right upper limb, and this indicates that they have good practice in this situation for saving the lives of their colleagues [12-13].

In spite of these results, the current study revealed that there is a significant increase of knowledge and attitudes regarding CPR among females rather than males in the majority of items and no significant difference between them in most practices. This indicates that the awareness of female students is better than that of males and similar results were find in other studies conducted in 2014 at an Ethiopian university and in 2015 in Hong Kong [13-14]. On the other hand, the present study shows that there are significant increases of knowledge, attitudes, and practices regarding CPR among medical students rather than non-medical students at Qassim University, and this is because their college curriculums will help them and increase their awareness regarding CPR, and there are no other studies done to support this results [13-14].

## Conclusions

Based on our study, the knowledge, attitudes, and practices of Qassim University students related to CPR are insufficient and need to be improved. Also, medical students are better than non-medical students and females are better at CPR than males so we recommend the incorporation of a BLS course, including CPR learning procedures, in the university curriculum, with regular reassessments, would increase the knowledge and application of CPR skills among students to save people lives.
